# IFITM1 suppresses expression of human endogenous retroviruses in human embryonic stem cells

**DOI:** 10.1002/2211-5463.12246

**Published:** 2017-06-29

**Authors:** Yudong Fu, Zhongcheng Zhou, Hua Wang, Peng Gong, Renpeng Guo, Jinmiao Wang, Xinyi Lu, Feng Qi, Lin Liu

**Affiliations:** ^1^ State Key Laboratory of Medicinal Chemical Biology College of Life Sciences Nankai University Tianjin China; ^2^ Department of Cell Biology and Genetics College of Life Sciences Nankai University Tianjin China; ^3^ Department of General Surgery Tianjin Medical University General Hospital China; ^4^ College of Pharmacy Nankai University Tianjin China

**Keywords:** H3K9me3, human embryonic stem cell, *IFITM1*

## Abstract

Interferon‐induced transmembrane protein 1 (IFITM1), a member of the IFITM protein family, is a component of a multimeric complex involved in the transduction of antiproliferation and cell adhesion signals. IFITM1 is thought to play a role in antiproliferation and immune surveillance, and has been shown to restrict infection by numerous viruses. It is highly expressed in human embryonic stem cells (hESCs) but its role in hESCs remains to be elucidated. In this study, knockout of *IFITM1* mediated by CRISPR/Cas9 in hESCs did not affect self‐renewal, pluripotency, telomerase activity or telomeres. However expression of human endogenous retroviruses (HERVs) was higher than in wild‐type hESCs, and there was also a reduced level of trimethylation of histone H3 on lysine 9 at HERV loci. These data show that IFITM1 suppresses HERVs in hESCs by regulating epigenetic modifications.

**Abbreviations**
ChIP: chromatin immunoprecipitation
CRC: colorectal cancer
ERV: endogenous retrovirus
H3K9me3: trimethylation of histone H3 on lysine 9
HEF: human embryonic fibroblast
HERV: human endogenous retrovirus
hESC: human embryonic stem cell
IFITM1: interferon‐induced transmembrane protein 1
LTR: long terminal repeat
PGC: primordial germ cell
qRT‐PCR: quantitative real‐time PCR
TERT: telomerase reverse transcriptase
TRF: terminal restriction fragment

Interferon‐induced transmembrane protein 1 (IFITM1), also known as interferon‐inducible protein 9–27, CD225 and Leu13, is a cell surface molecule that is important for antiproliferative and homotypic adhesion signal transduction in lymphocytes 1, 2. IFITM1 could be highly induced by interferon‐α and ‐γ in response to infection by pathogens, and demonstrated antiviral activities, such as inhibition of influenza A replication and enveloped virus infection 3. Moreover, IFITM proteins restrict infection of various types of viruses by suppressing viral membrane fusion and infection before the occurrence of hemifusion, through interruption of viral coreceptor function or inhibition of virus entry and replication 4, 5, 6, 7.

IFITM1 expression was found to be elevated in cancers of the cervix 8, esophagus 9, ovary 10 and brain 11. Overexpression of IFITM1 has clinicopathological effects on gastric cancer and is regulated by an epigenetic mechanism 12. Furthermore, IFITM1 is up‐regulated in human colorectal cancer (CRC) and has been identified as a molecular marker in human colorectal tumors 13. Recently, IFITM1 was found to be highly expressed in metastatic CRC cell lines as well as colorectal patient‐derived tumor samples, and its high expression is associated with a poor prognosis of the disease 14, 15, or a more advanced clinical stage 16.

Interestingly, IFITM1 is also expressed in mouse primordial germ cells (PGCs) and is implicated in PGC development 17, and in human naïve pluripotent stem cells 18. However, it also has been shown that *Ifitm* genes are not essential for PGC migration and the *Ifitm* family appears to be functionally redundant during development 19. The function and implication of IFITM1 expression in pluripotent stem cells remain unclear. Here we investigated the potential roles and the underlying mechanisms of IFITM1 in human embryonic stem cell (hESCs).

## Materials and methods

### Cell culture

Human embryonic stem cells (RUES2 WT and RUES2 IFITM1‐KO) were cultured at 37 °C in 5% CO_2_ in Essential 8 medium (A1517001, Life Technologies, Carlsbad, CA, USA). Human embryonic fibroblast (HEF) cells were cultured in Dulbecco's modified Eagle's medium, 10% FBS, 1% l‐glutamine and 1% penicillin/streptomycin.

### Immunofluorescence

Cells were washed twice in phosphate buffered saline (PBS), then fixed in freshly prepared 3.7% paraformaldehyde in PBS for 15 min on ice, washed three times, permeabilized in 0.1% Triton X‐100 in blocking solution (3% goat serum plus 0.5% BSA in PBS) for 30 min at room temperature, washed three times each for 5 min, and left in blocking solution (3% goat serum plus 0.5% BSA in PBS) for 2 h. Cells were incubated overnight at 4 °C with primary antibodies against IFITM1/2/3 (F12; sc‐374026; Santa Cruz Biotechnology, Dallas, TX, USA), IFITM3 (AF3377; R&D Systems, Minneapolis, MN, USA), OCT3/4 (SC‐5279; Santa Cruz Biotechnology), or 53BP1 (ab36823, Abcam, Cambridge, MA, USA), washed three times and incubated for 1.5 h with secondary antibodies (goat anti‐mouse IgG (H + L) fluorescein isothiocyanate, 115‐095‐003, Jackson ImmunoResearch Laboratories, West Grove, PA, USA; goat anti‐rabbit IgG (H + L) Alexa Fluor®, 594A‐11037, Life Technologies) at room temperature, diluted 1 : 200 with blocking solution. Samples were washed, and counterstained with 5 μg·mL^−1^ 4′,6‐diamidino‐2‐phenylindole (DAPI) in Vectashield mounting medium. Fluorescence was detected and imaged using a motorized fluorescence microscope (Zeiss Axio Imager Z1; Carl Zeiss, Jena, Germany).

### Knockout of IFITM1 by CRISPR/Cas9

pSpCas9 (BB)‐2A‐Puro (PX459) was a gift from Feng Zhang (Addgene plasmid no. 48139). Guide RNAs were designed using the online design tool available at http://crispr.mit.edu. PX459 was digested with *Bbs*I and then gel purified. Pairs of oligos including targeting sequences were annealed and cloned into the *Bbs*I‐digested PX459 vector. The primers are listed in Table S1. CRISPR plasmid was nucleofected into RUES2 human ESC using Human Stem Cell Nucleofector Kit 1 (VPH‐5012; Lonza, Basel, Switzerland) by Amaxa Nucleofector II (Program B‐016; Lonza). 24 h after nucleofection, 0.4 μg?mL^−1^ puromycin was added into culture medium for 12 h, and single cell clone was selected after limited dilution. Clones were genotyped by PCR and T7E1 assay, verified by sequencing.

### Gene expression analysis by quantitative real‐time PCR

Total RNA was purified using an RNA Mini Kit (Qiagen, Hilden, Germany) for cells, treated with DNase I (79254; Qiagen). RNA was subject to cDNA synthesis using M‐MLV Reverse Transcriptase (28025021, Life Technologies). The PCR reaction was set up in duplicate using the FastStart Universal SYBR Green Master (4913914001; Roche, Basel, Switzerland) and run on the Realplex PCR detection system (Bio‐Rad Laboratories, Hercules, CA, USA) using primer sets specific for each gene. Primers were designed using PrimerQuest Tool provided by the Integrated DNA Technologies website (http://www.idtdna.com/site) or based on previous publications and confirmed for their specificity by dissociation curves. All reactions (in duplicate) were carried out by amplifying target genes and endogenous glyceraldehyde 3‐phosphate dehydrogenase as control in the same plate. Relative quantitative evaluation of target genes was determined by comparing the threshold cycles. cDNA was used as the template for quantitative real‐time PCR (qRT‐PCR) amplification using the primers in Table S3.

### Western blot

The western blot experiment was performed as described previously 20 and the antibodies used were: IFITM1/2/3 (F12; sc‐374026; Santa Cruz Biotechnology), NANOG (sc‐293121; Santa Cruz Biotechnology), OCT3/4 (SC‐5279; Santa Cruz Biotechnology), SOX2 (AB5603, Millipore, Billerica, MA, USA), β‐actin (sc1616R; Santa Cruz Biotechnology) and IFITM3 (AF3377; R&D Systems). The protein bands were detected by Amersham ECL Prime western blot detection reagent (RPN2232; GE Healthcare, Chicago, IL, USA).

### Telomere measurement by quantitative real‐time PCR

Genomic DNA from the cell lines was isolated with DNeasy Blood & Tissue kit (69504; Qiagen). The DNA quality was assayed using a Nanodrop 2000 spectrophotometer, and the ratio of 260–280 nm was between 1.8 and 2.1. Average telomere length was measured using real‐time PCR assay, as previously described 21. PCR reactions were performed on the Realplex PCR detection system (Bio‐Rad Laboratories). For each PCR reaction, a standard curve was made by serial dilutions of known amounts of DNA from human fibroblast cells. The telomere signal (T) was normalized to the signal from the single copy gene (S) human 36B4 to generate a T/S ratio indicative of relative telomere length. Each sample was repeatedly measured at least three times. Primers for the T/S ratio 22 are listed in Table S2.

### Telomere terminal restriction fragment analysis by Southern blot analysis

Telomere terminal restriction fragment (TRF) analysis was performed using a commercial kit (TeloTAGGG Telomere Length Assay, 12209136001 ;Roche Life Science). Genomic DNA from the cell lines was isolated with DNeasy Blood & Tissue kit (69504; Qiagen), and 1.5 μg DNA for each sample was digested using *Hin*fI and *Rsa*I restriction enzymes. Digested DNA underwent electrophoresis through a 0.8% agarose gel (111860; Biowest, Nuaille, Maine‐et‐Loire, France) for 4 h at 6 V·cm^−1^ in 1× Tris/acetate/EDTA (TAE) buffer. Gels were denatured, neutralized and transferred to positively charged nylon membranes (RPN2020B, GE Healthcare) overnight. The membranes were hybridized in DIG Easy Hyb Granules (11796895001; Roche Diagnostics, Penzberg, Germany) containing the telomere probe at 42 °C overnight. The TRF length was quantitatively measured according to the kit instructions.

### Cell cycle analysis

Cells (WT and IFITM1‐KO RUES2 cells) were fixed in freshly prepared 70% ethanol at 4 °C overnight, then centrifuged at 1000 ***g*** for 5 min to collect cells and stained with propidium iodide at 37 °C for 30 min in a water bath. Fluorescence‐activated cell sorting analysis was used to determine cell cycle phases.

### Chromatin immunoprecipitation–qPCR

Chromatin immunoprecipitation (ChIP)–qPCR was performed based on a published protocol 23. Chromatin extracts were immunoprecipitated using trimethylation of histone H3 on lysine 9 (H3K9me3; ab8898 Abcam) antibody. Input and immunoprecipitation samples were analyzed by qPCR and the primers are listed in Table S3.

### Telomerase activity assay

Telomerase activity was determined by the Stretch PCR method according to the manufacturer's instructions using the TeloChaser Telomerase assay kit (T0001; MD Biotechnology, Xiamen, China). About 2.5 × 10^4^ cells from each sample were lysed, and lysed cells heated at 70 °C for 10 min served as negative control. PCR products of cell lysates were separated on non‐denaturing TBE‐based 10% polyacrylamide gel electrophoresis and visualized by ethidium bromide staining.

### Statistical analysis

Data were analyzed by ANOVA and means compared by Fisher's protected least‐significant difference (PLSD) using statview software (SAS Institute Inc., Cary, NC, USA). Significant differences were defined as *P *<* *0.05, 0.01 or lower.

## Results

### Establishment of IFITM1‐knockout hESC lines

**Figure 1:**
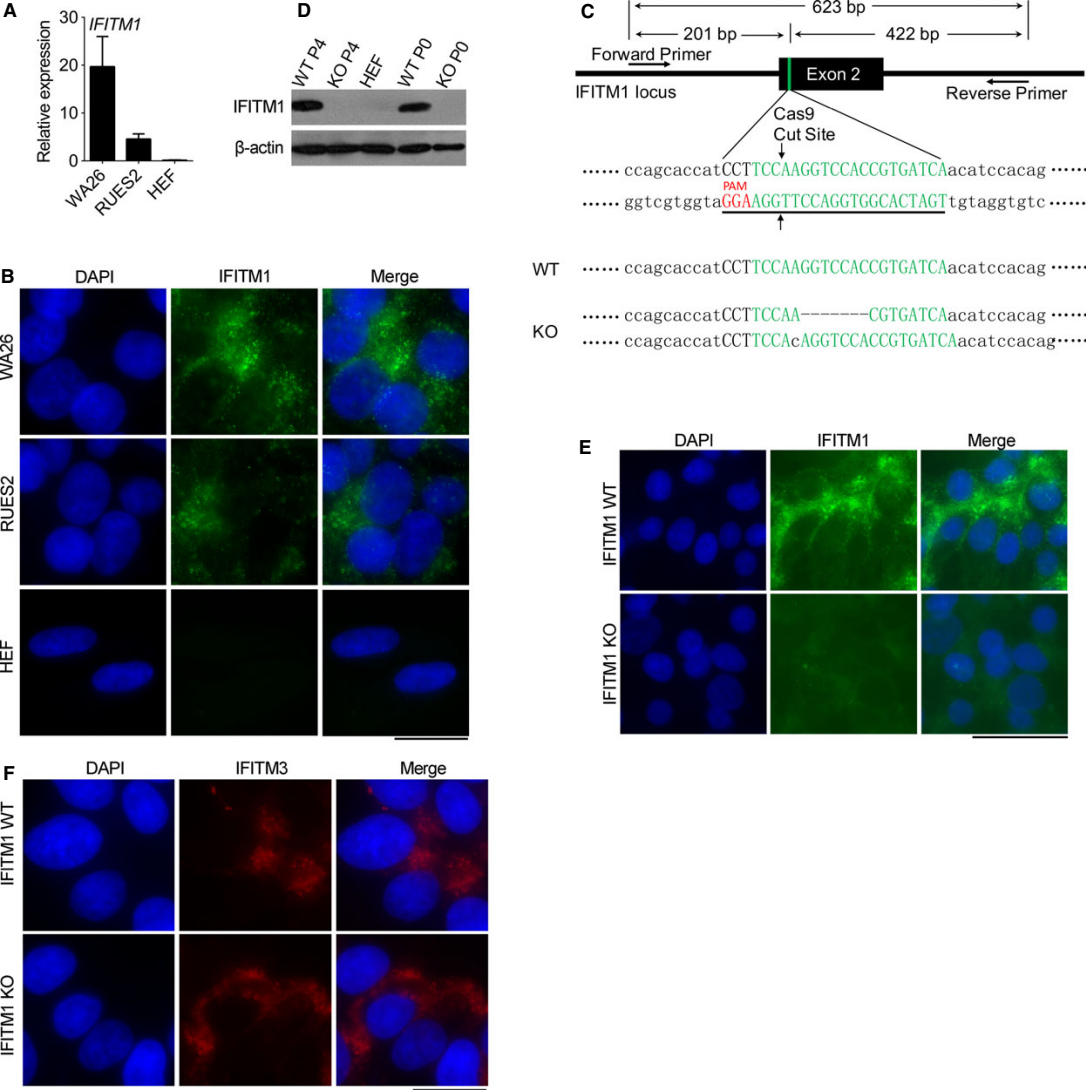
Establishment of *IFITM1*‐knockout hES cell lines. (A) Expression levels of *IFITM1* by qRT‐PCR in hESC lines (RUES2 and WA26) and HEF. Data represent the means and SEM (*n *=* *4). (B) Representative immunofluorescence of IFITM1 in hESC lines (WA26 and RUES2) and HEF cells; HEF cells served as positive control. Nuclei were counterstained by DAPI. Scale bar: 20 μm. (C) Schematic diagram of CRISPR/Cas9‐mediated *IFITM1* knockout in hESC lines. (D) Western blot analysis showing the acquisition of *IFITM1* KO hESC lines. HEF cells served as positive control, and β‐actin as loading control. (E) Representative immunofluorescence microscopic images of IFITM1 in hESC at P0. Scale bar: 50 μm. (F) Representative immunofluorescence microscopic images of IFITM3 in hESC at P0. Scale bar: 20 μm.

We analyzed mRNA expression levels of IFITM1 by qRT‐PCR in hESCs (WA26 and RUES2) that expressed notably higher mRNA levels of IFITM1 than did human fibroblast cells (HEF; Fig. 1A). Also we performed immunofluorescence microscopy of hESCs (WA26 and RUES2) and HEFs. Notably, IFITM1 was localized on the cell surface and cytoplasm and expressed at higher levels in hESCs than HEFs (Fig. 1B). To explore the potential role of IFITM1, we took advantage of CRISPR/Cas9 technology and generated IFITM1‐knockout hESCs (Fig. 1C). Both western blot and immunofluorescence validated that IFITM1 protein was undetectable in IFITM1 KO hESCs, comparable to that of HEFs, in contrast to WT hESCs (Fig. 1D,E). IFITM1 antibody may also detect IFITM3 due to similarity of their protein sequences. Therefore, we conducted immunofluorescence staining for IFITM3 in IFITM1 KO and WT hESCs to test the specificity of the sgRNAs used in the CRISPR/Cas9 method. IFITM3 was found in both IFITM1 KO and WT hESCs by immunofluorescence (Fig. 1F), and there was no difference of IFITM3 levels between IFITM1 KO and WT hESCs shown by western blot (Fig. S1), further supporting that the designed sgRNAs were specific to IFITM1, consistent with the gene sequencing data.

### Effects of IFITM1 knockout on pluripotency and telomere length in hESCs

**Figure 2:**
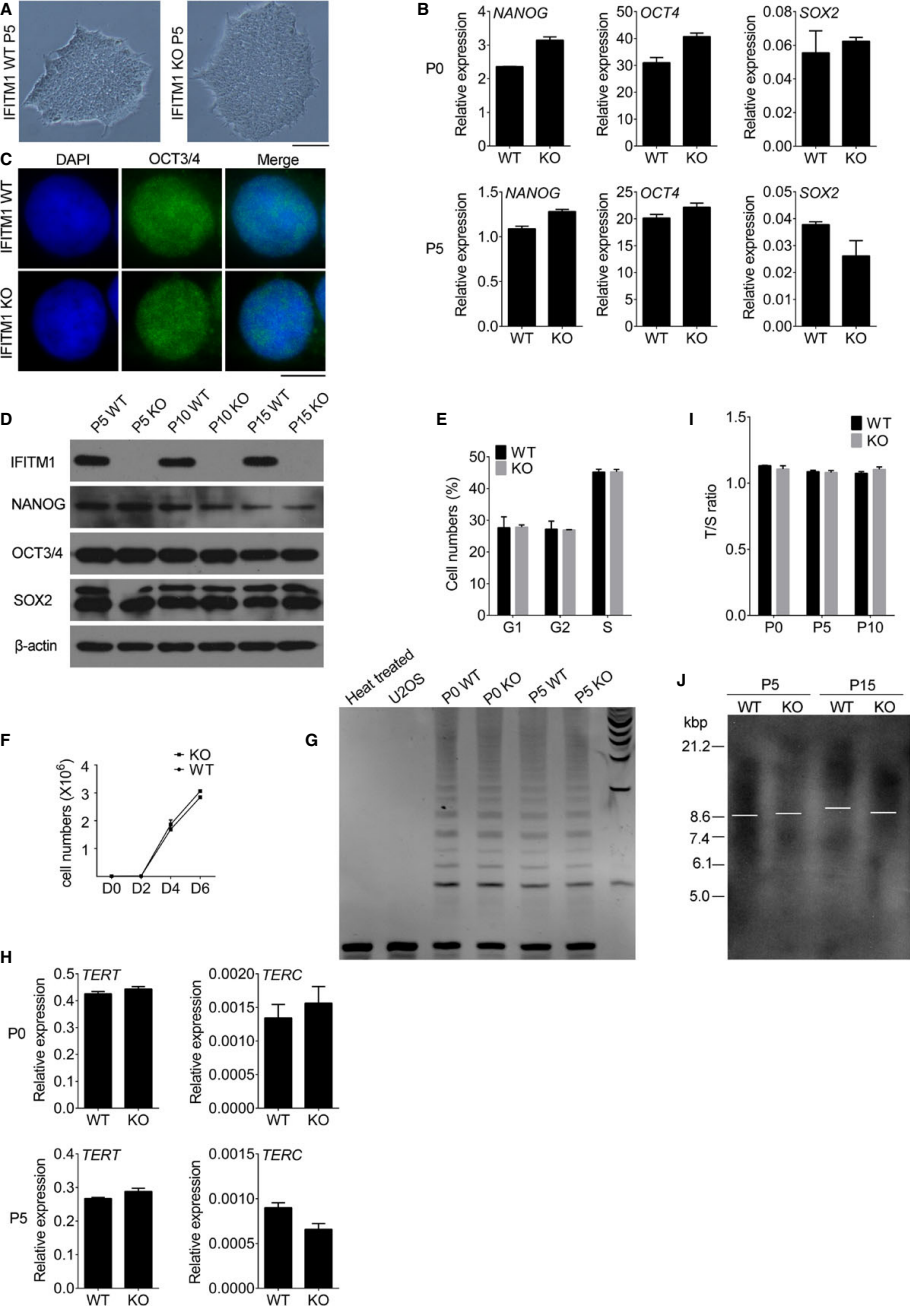
Impact of *IFITM1*‐knockout on pluripotency and telomere length in hESCs (RUES2). (A) The cell morphology of *IFITM1* KO and WT hESCs at P5. (B) Expression levels of *NANOG*,*OCT4*, and *SOX2* by qRT‐PCR in *IFITM1* KO and WT hESCs (RUES2) at P0 and P5. Data represent the mean and SEM (*n *=* *4). (C) Representative immunofluorescence microscopic images showing OCT3/4 expression in *IFITM1* KO and WT hESCs at P5. Scale bar: 10 μm. (D) Western blot analysis of protein levels of NANOG, OCT3/4 and SOX2 in *IFITM1* KO and WT hESCs at P5, P10 and P15 and β‐actin as loading control. (E) Cell number during cell cycle progression in *IFITM1* KO and WT hESCs at P5. Two repeats. (F) Cell proliferation determined by number of cells in *IFITM1* KO and WT hESCs. The growth curve of the RUES2 *IFITM1* KO and WT hESCs at P5. Cells were seeded (1000 cells per well) 24 h prior to counting, and counted every 24 h. Two repeats. (G) Telomerase activity by telomeric repeat amplification protocol in *IFITM1* KO and WT hESCs at P0 and P5. (H) Expression levels of *TERT* and *TERC* by qPCR in *IFITM1* KO and WT hESCs at P0 and P5. Data represent the mean and SEM (*n *=* *4). (I) Telomere lengths do not differ between WT and *IFITM1* KO hESCs, shown as T/S ratio by qPCR. Data represent the mean and SEM (*n *=* *4). (J) Telomere lengths do not differ between WT and *IFITM1* KO hESCs, shown as TRF by Southern blot.

ESC colonies were similar in morphology between IFITM1 KO and WT hESCs (Fig. 2A). To elucidate whether high expression level of IFITM1 is required for pluripotency of hESCs, we examined whether the mRNA levels of *OCT4*,* NANOG* and *SOX2*, which are important for pluripotency, were similar between IFITM1 KO and WT hESCs (Fig. 2B). Also protein levels of NANOG, OCT4 and SOX2 by western blot did not differ between IFITM1 KO and WT hESC lines at various passages (Fig. 2D). In addition, OCT4 immunofluorescence was similar between IFITM1 KO and WT hESCs (Fig. 2C).

Cell proliferation, which was determined by the cell number and cell cycle progression, also did not differ between IFITM1 KO and WT hESCs (Fig. 2E,F). Telomerase is critical for cell proliferation and is a complex of reverse transcriptase comprising two core components: Telomerase reverse transcriptase (*TERT*) and template RNA *TERC* (essential RNA component) 24. Consistent with cell proliferation, IFITM1 deficiency did not alter telomerase activity and the expression levels of *TERT* and *TERC* (Fig. 2G,H). Telomere length is primarily maintained by telomerase and cell dividing times and predicts replicative capacity 25. Also, IFITM1 KO and WT hESCs at early or late passages presented similar telomere lengths shown as T/S ratio by qPCR (Fig. 2I), and also determined by TRF (Fig. 2J). Besides we performed immunofluorescence analysis of the DNA damage response marker 53BP1 26 in IFITM1 KO and WT hESCs (Fig. S2A). The number of 53BP1 positive cells and 53BP1 foci number per cell in IFITM1 KO and WT hESCs were quantified, and increased in IFITM1 KO hESCs compared with WT hESCs (Fig. S2B,C).

### Expression of human endogenous retroviruses in hESCs and epigenetic regulation by IFITM1

**Figure 3:**
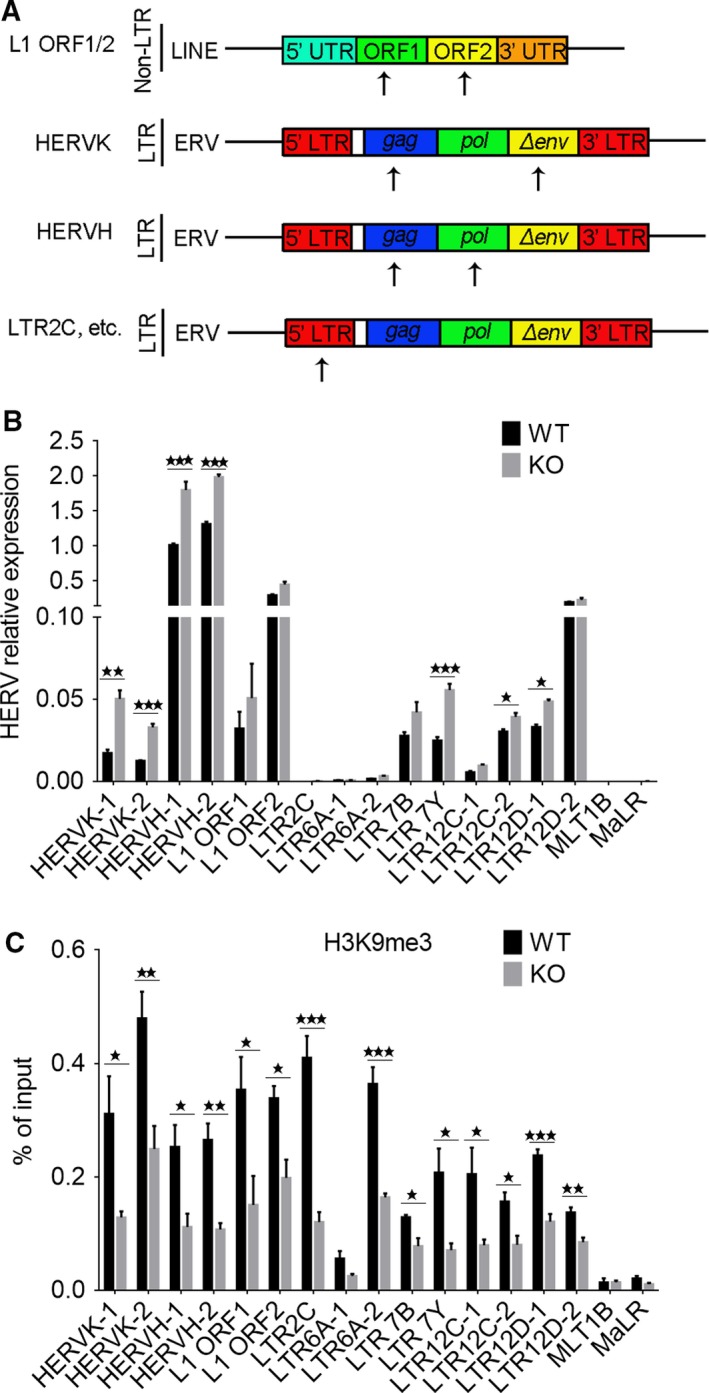
Expression of HERV *s* in *IFITM1* knockout hESCs compared with WT hESCs. (A) Schematic of non‐LTR retrotransposons including 5′ and 3′ UTR and ORF1 and ORF2, and ERVs that have 5′ and 3′ LTRs, and an ‘internal’ region that includes retroviral ORFs gag, pol and env. The black arrow indicates the region of primers used for qPCR. (B) Expression levels of HERVs by qPCR of *IFITM1*‐knockout hESCs at passage 15. Data represent the mean and SEM (*n *=* *4). (C) Enrichment of H3K9me3 at various HERVs by ChIP‐qPCR using primers for the region of the HERVs at P15. Data represent mean and SEM (*n *=* *4). *P <* *0.05; **P <* *0.01; ***P <* *0.001.

Endogenous retroviruses (ERVs) are transposable genetic elements that comprise nearly 8% of the human genome 27, and can copy and paste their own DNA into the genome 28, 29. ERVs are activated during embryonic development and inactivated during ESC isolation and culture 18. IFITM1‐mediated restriction may be an evolutionarily conserved mechanism protecting both embryos and germ cells from either reinfection of infectious ERVs or exogenous viral infection 18. We asked whether IFITM1 regulates ERVs in hESCs by examining relative expression of ERVs in IFITM1 KO and WT hESCs (RUES2) using specific primers (Table S3) 30. With increasing passages (by P15), *HERVK*,* HERVH, LTR7Y* and *LTR12D‐1* were highly up‐regulated in IFITM1 KO hESCs, compared with WT hESCs (Fig. 3A,B). Human endogenous retroviruses (HERVs), especially *HERVH*, are expressed preferentially in hESCs 31, and these elevated further in IFITM1 KO hESCs.

Endogenous retrovirus containing long terminal repeats (LTRs) are silenced through H3K9me3 by ERG‐associated protein with SET domain (ESET; also known as SETDB1 or KMT1E) in mouse ESCs 32. We tested whether H3K9me3 regulates HERV expression through analysis of the level of H3K9me3 on HERVs by ChIP‐qPCR. Enrichment of H3K9me3 was reduced at *HERVK*,* HERVH, LTR7Y* and *LTR12D‐1/2* in IFITM1 KO hESCs compared with WT ESCs (Fig. 3C). Decreased levels of H3K9me3 at these HERV foci could partly explain the elevated expression of HERVs in the IFITM1 KO hESCs, but how IFITM1 reduces H3K9me3 enrichment at HERVs remains to be determined.

## Discussion

*IFITM* family members were described as interferon‐induced genes, and they are also classical naive‐state and PGC markers in the mouse, which nonetheless appear to be dispensable for development 19. IFITM1 has an essential part in regulating viral infection 33. It was found to be present as a tight junction protein induced by type 1 interferon in hepatocytes, and it acts by interacting with viral coreceptors to prevent viral entry into cells 34. Tight junction proteins can interact with adapter proteins and subsequently mediate cell signaling pathways and transcription 35, 36. Since IFITM1 is a tight junction protein, it is likely that it may act by cooperating with other membrane proteins to activate or repress downstream regulators and in turn affect the epigenetic status of ERVs as well. IFITM1 could also be activated by ERV expression. Expression of *HERVK* in pluripotent cells could further precisely activate IFITM1 and restrict viral infection, but many other interferon‐induced genes are not upregulated or expressed 18. For example, we found the expression of IFITM3 was not upregulated in IFITM1 KO hESCs in which HERVs were increased, suggesting a feedback loop between HERV expression and IFITM1‐mediated defense of viral infection and also suggesting that other IFITM proteins may not respond to the upregulated HERVs, though we could not state that they do not regulate HERVs in hESCs. Interestingly, we found that knockout of IFITM1 further promotes the expression of HERVs in hESCs by reducing the level of H3K9me3 at HERV loci, although it has minimal impact on cell proliferation and pluripotency. HERVs, such as *HERVH*, play an important role in maintaining pluripotency in hESCs 37, 38. Overexpression of HERVs could result in upregulation of adjacent genes and might link to unbalanced chromosomal translocations 39; aberrant expression of HERVs can lead to cancer, which is also viewed as a ‘genomic disease’ 14. We found that DNA damage was increased in IFITM1 KO hESCs in which HERVs were increased, which suggests that IFITM1 plays a role in repressing excessive activation of HERVs and thus may play important roles in genome maintenance. Together, our data suggest that IFITM1 may participate in suppressing HERVs by regulating level of H3K9me3 at HERV loci in hESCs, which may maintain stability of hESCs. It suggests that IFITM1‐mediated restriction may be an evolutionarily conserved mechanism protecting cells from reinfection from infectious ERV infection.

## Author contributions

YDF designed and conducted the most experiments and analyzed data. ZCZ, HW, PG, RPG and JMW performed some experiments and provided technical support. XYL discussed and interpreted results and revised the manuscript. FQ and LL interpreted results and wrote and revised the manuscript.

## Supporting information


**Table S1.** Primers used for knockout of IFITM1 by CRISPR/Cas9 system.
**Table S2.** Primers for T/S ratio.
**Table S3.** Primers for qRT‐PCR and ChIP‐qPCR.
**Fig. S1.** Impact of IFITM1‐knockout on protein level of IFITM3.
**Fig. S2.** Impact of IFITM1‐knockout on DNA damage.
