# Supplementary material for: IFITM1 suppresses expression of human endogenous retroviruses in human embryonic stem cells
Source: FEBS Open Bio. 2017 Jun 29;7(8):1102–10. doi: 10.1002/2211-5463.12246 (PMC5537067; doi:10.1002/2211-5463.12246)
Supplement: Supplementary file 1 — Table S1. Primers used for knockout of IFITM1 by CRISPR/Cas9 system. Table S2. Primers for T/S ratio. Table S3. Primers for qRT‐PCR and ChIP‐qPCR. Fig. S1. Impact of IFITM1‐knockout on protein level of IFITM3. Fig. S2. Impact of IFITM1‐knockout on DNA damage. [file FEB4-7-1102-s001.doc]

**Supplementary Figures**


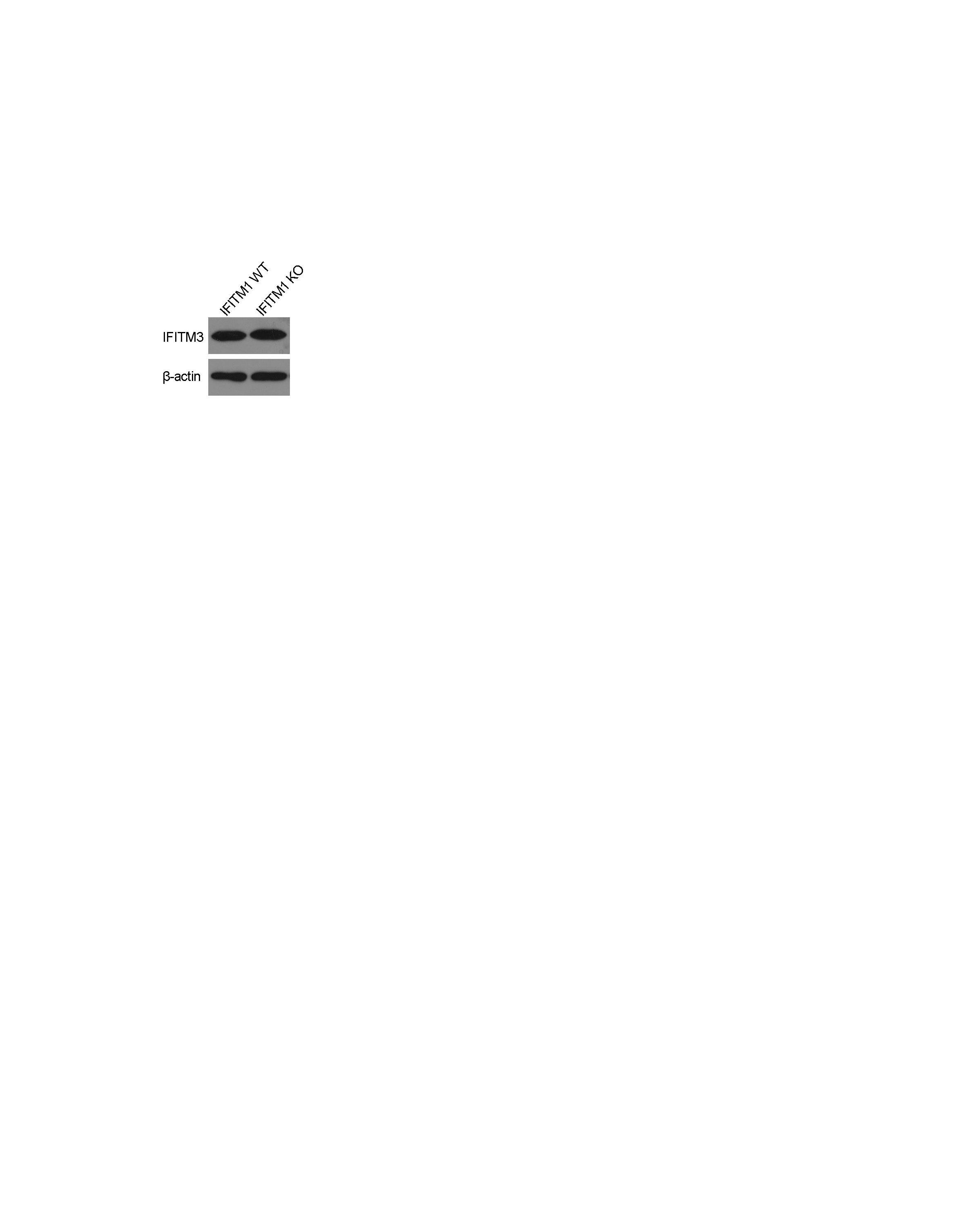


**Fig. S1. Impact of *IFITM1*-knockout on protein level of IFITM3.** Western blot analysis of protein levels of IFITM3 in *IFITM1* KO and WT hESCs at P15 and β-actin as loading control.

**
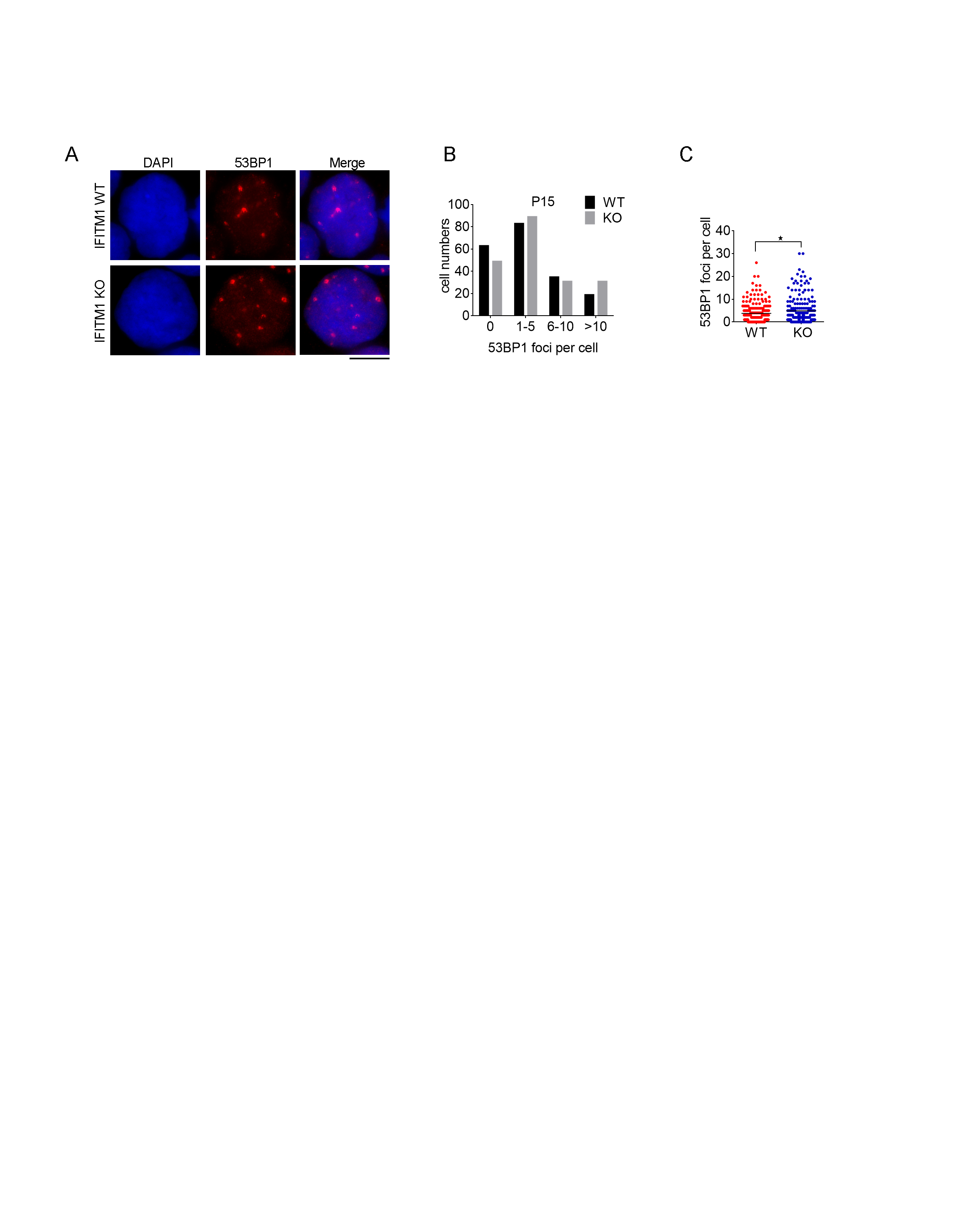
**

**Fig. S2. Impact of *IFITM1*-knockout on DNA damage.**

(A) Representative immunofluorescence microscopic images showing 53BP1 expression in *IFITM1* KO and WT hESCs at P15. Scale bar = 10 μm. (B) The number of 53BP1 positive cells in *IFITM1* KO and WT hESCs at P15. (C) The number of 53BP1 foci per cell in *IFITM1* KO and WT hESCs at P15 (n=200 cells). The black line indicates the average value. *, *P<*0.05.

**Supplementary Tables**

**Supplementary Table 1. Primers used for Knockout of *IFITM1*** by CRISPR/Cas9 system

|  | Forward(5’ to 3’) | Reverse (3’ to 5’) |
| --- | --- | --- |
| hIFITM1-Oligo-sgRNA | CACCGTGATCACGGTGGACCTTGGA | AAACTCCAAGGTCCACCGTGATCAC |

**Supplementary Table 2. Primers for T/S ratio**

|  | Forward(5’ to 3’) | Reverse(3’ to 5’) |
| --- | --- | --- |
| Telomere | CGGTTTGTTTGGGTTTGGGTTTGGGTTTGGGTTTGGGTT | GGCTTGCCTTACCCTTACCCTTACCCTTACCCTTACCCT |
| h36B4 | CAGCAAGTGGGAAGGTGTAATCC | CCCATTCTATCATCAACGGGTACAA |

**Supplementary Table 3.Primers for qRT-PCR and ChIP-qPCR**

|  | Forward(5’ to 3’) | Reverse(3’ to 5’) |
| --- | --- | --- |
| GAPDH | TCGACAGTCAGCCGCATCTTCTTT | ACCAAATCCGTTGACTCCGACCTT |
| IFITM1 | ACTCCGTGAAGTCTAGGGACA | TGTCACAGAGCCGAATACCAG |
| TERT | AGCACCGTCTGCGTGAG | CAGCTCGACGACGTACACAC |
| TERC | GGTGGTGGCCATTTTTTGTC | CTAGAATGAACGGTGGAAGGC |
| OCT4 | GACAGGGGGAGGGGAGGAGCTAGG | CTTCCCTCCAACCAGTTGCCCCAAAC |
| NANOG | TTTGGAAGCTGCTGGGGAAG | GATGGGAGGAGGGGAGAGGA |
| SOX2 | CAAAAATGGCCATGCAGGTT | AGTTGGGATCGAACAAAAGCATT |
| HERVK-1 | GCTGCCCTGCCAAACCTGAG | CCTGAGTGACATCCCGCTTACC |
| HERVK-2 | AAATAAGACCCAACCGCCAGTAGC | GAATTGCCATGCCTCAGTATCTCC |
| HERVH-1 | GCCTCTGCTCCTCCACCCTATA | GTTGCTGCCAAACGAGCCATGA |
| HERVH-2 | CCTTTTCTACAGACCCATCTGA | GCATTAACCTTGACTATGCGTT |
| L1 ORF1 | AGAAATGAGCAAAGCCTCCA | GCCTGGTGGTGACAAAATCT |
| L1 ORF2 | CCACCGATCCCACAGAAATA | TGGTCCTGGACTCTTTTTGG |
| LTR2C | GAGACTTGAGTCCGCTGAAGCT | CTGCCCACCTTCTCTGACTC |
| LTR6A-1 | CCAAGAATGCAATGAACTGGTA | CAGTTCCAGGTACAGGAGCTTT |
| LTR6A-2 | GCTCCTGTACCTGGAACTGTTT | TACCGAGACAGCTAGTGCTCAA |
| LTR7B | CTTTGTAATTCTCCCCACCCTTGAG | GGATTATCATTAGTTCTTATAGGTT |
| LTR7Y | TATACATCCAGATGGCCTGAAGT | GTGGGGAGAATTACAAAGAACCT |
| LTR12C-1 | CATCTGGAGCTGTTCTTTCCTC | GTGGGGCCAGATAAGAGAATAA |
| LTR12C-2 | CCAAAGATTGAGTAGCAGCAAG | CTCTACCAATCAGCAGGATGTG |
| LTR12D-1 | GACCAATCAGCTCTCTGTAAAATG | TGCCTGCGTTTATTCCCTTATCTGG |
| LTR12D-2 | GATCTGACGCTTCACTCCTGAAGTCAG | TCCTTCTGGTGGGTTCCTGGTCT |
| MLT1B | GATCAGGGCCCACTCTATTTAT | GCATCCTGCAAACGAACTGACTA |
| MaLR | CCTCAGAGTGCCTTCCATTTTGTTA | CTCACCAGGCCCCTCCTCCAA |

HERVK-1：Name: HERVK-int, Family: ERVK, Class: LTR

HERVK-2：Name: HERVK-int, Family: ERVK, Class: LTR

HERVH-1：Name: HERVH-int, Family: ERV1, Class: LTR

HERVH-2：Name: HERVH-int, Family: ERV1, Class: LTR

L1 ORF1:Name: L1HS, Family: L1, Class: LINE

L1 ORF2:Name: L1PA3, Family: L1, Class: LINE

LTR2C:Name: LTR2C, Family: ERV1, Class: LTR

LTR6A-1:Name: LTR6A, Family: ERV1, Class: LTR

LTR6A-2: Name: LTR6A, Family: ERV1, Class: LTR

LTR7B:Name: LTR7B, Family: ERV1, Class: LTR

LTR7Y:Name: LTR7B, Family: ERV1, Class: LTR

LTR12C-1:Name: LTR12C, Family: ERV1, Class: LTR

LTR12C-2:Name: LTR12C, Family: ERV1, Class: LTR

LTR12D-1:Name: LTR12D, Family: ERV1, Class: LTR

LTR12D-2:Name: LTR12D, Family: ERV1, Class: LTR

MLT1B:Name: MLT1B, Family: ERVL-MaLR, Class: LTR

MaLR:Name: MSTA,Family: ERVL-MaLR, Class: LTR
